# An integrated approach for analyzing spatially resolved multi-omics datasets from the same tissue section

**DOI:** 10.3389/fmolb.2025.1614288

**Published:** 2025-07-15

**Authors:** Thao Tran, Felicia Wee, Craig Ryan Joseph, Wanqiu Zhang, Jeffrey Chun Tatt Lim, Zhen Wei Neo, Li Yen Chong, Francis Hong Xin Yap, Nathan Heath Patterson, Marc Claesen, Alice Ly, Joe Yeong

**Affiliations:** ^1^ Aspect Analytics NV, Genk, Belgium; ^2^ Institute of Molecular and Cell Biology (IMCB), Agency for Science, Technology and Research (A*STAR), Singapore, Singapore; ^3^ Department of Anatomical Pathology, Singapore General Hospital, Singapore, Singapore; ^4^ Department of Microbiology, Immunology and Serology Section, Singapore General Hospital, Singapore, Singapore

**Keywords:** spatial multi-omics, image registration, single cell analysis, spatial transcriptomics, spatial proteomics, histology, data integration

## Abstract

Recent advances in spatial transcriptomics (ST) and spatial proteomics (SP) technologies have enabled high-dimensional molecular profiling at single-cell resolution, providing deeper insights into the tumour-immune microenvironment. However, these modalities are typically applied to separate tissue sections, limiting direct comparisons across molecular layers. We developed a wet-lab and computational framework to perform and integrate ST and SP from the same tissue section, as demonstrated on human lung cancer samples. Applying ST, SP, and hematoxylin and eosin (H&E) staining from the same section ensured consistency in tissue morphology and spatial context. Computational registration using Weave software allowed accurate alignment and annotation transfer across modalities. This co-registered dataset enabled single-cell level comparisons of RNA and protein expression, revealed segmentation accuracy and transcript-protein correlation analyses within individual cells. Notably, we observed systematic low correlations between transcript and protein levels—consistent with prior findings—now resolved at cellular resolution. Our approach highlights the feasibility and utility of performing spatially-resolved multi-omics analysis on the same section without compromising data quality, facilitating concordance studies and region-specific analysis of immune and tumour markers, and ultimately advancing our understanding of disease heterogeneity at the molecular level.

## 1 Introduction

Single-omics spatial technologies have transformed our understanding of disease by enabling spatially resolved insights across genomic, transcriptomic, metabolomic, and proteomic layers, offering an unprecedented view into cellular organization and interactions within tissue contexts (Rivest et al., 2023; Goltsev et al., 2018; Janesick et al., 2023; He et al., 2022). However, each omics modality captures only a partial aspect of the complex biological landscape. Many of their discoveries remain isolated (Baysoy et al., 2023; Lim et al., 2024), limiting our ability to fully grasp cancer heterogeneity and cell-to-cell interactions that drive disease progression (Chen et al., 2020; Chakraborty et al., 2024).

To overcome this limitation, spatially resolved multi-omics has emerged as a powerful approach to integrate multiple spatial technologies to uncover deeper biological insights through cross-modal correlation. Recent studies have demonstrated that multimodal spatial analyses can reveal novel signalling pathways, refine therapeutic biomarkers, and offer insights beyond what single-modality approaches can achieve (Senosain et al., 2023; Asada et al., 2024). Yet, despite its potential, the practical application of multi-omics remains constrained by the technical challenges of data generation, integration, and analysis. A major bottleneck is that spatial multi-omics data is typically collected from adjacent tissue sections due to differences in sample preparation protocols, which introduces spatial misalignment and complicates direct cell-to-cell comparisons. Additionally, integrating multi-omics data involves challenges for preprocessing (Zhang Y. et al., 2024), normalization, and integration across platforms (Mohr et al., 2024), amongst others.

A key missing component in spatial multi-omics research is the ability to simultaneously profile mRNA and protein expression at high spatial resolution within the same tissue section. While a few recent studies have explored this direction (Liao et al., 2023; Rademacher et al., 2024), there is still a lack of systematic approaches demonstrating how transcriptomic and proteomic data can be jointly leveraged at the single-cell level for biological discovery.

**FIGURE 1:**
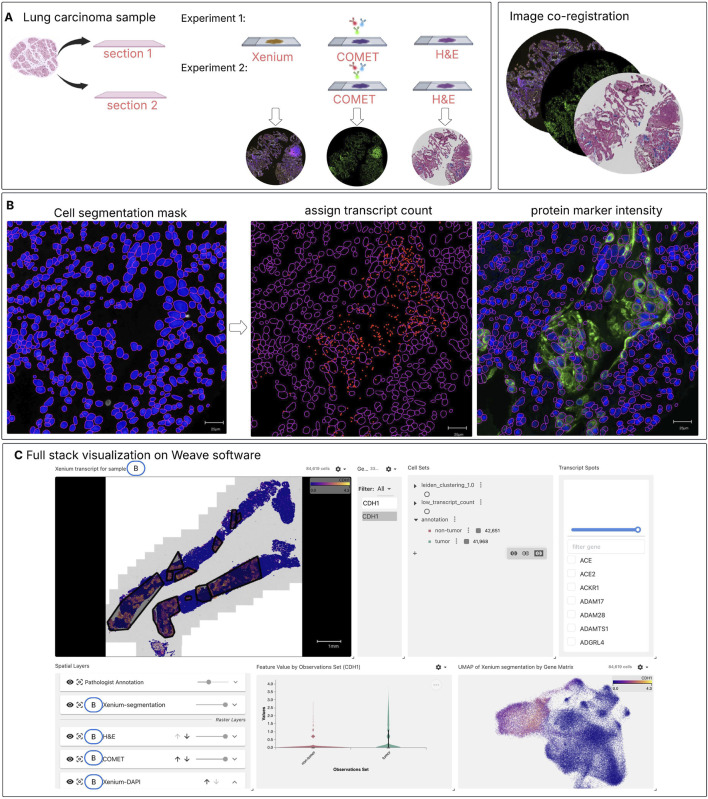
Spatial multi-omics workflow for integrating spatial proteomics (SP) and spatial transcriptomics (ST) on the same tissue section. **(A)** Two consecutive lung carcinoma tissue sections were analyzed under different experimental conditions: Experiment 1 —processed sequentially with Xenium, COMET, and H&E staining and Experiment 2— without Xenium, processed sequentially with COMET and then H&E staining. The Xenium and COMET data were co-registered to the H&E image (post-Xenium, post-COMET) using an automated non-rigid registration. **(B)** After alignment, cell segmentation masks (here segmentation mask from protein markers is used for demonstration) are applied to associate captured transcript spots with individual cells and to calculate the mean intensity of protein markers per cell. This integration produces a unified dataset that includes both transcript counts and protein marker intensities within the same cells**. (C)** An interactive web-based visualization was created in Weave, integrating all modalities—including tumour annotations (gray mask) from the H&E-stained section, cell segmentation, and transcript spots. This enables users to explore and examine downstream analysis results across modalities, such as correlation analyses within tumour and non-tumour regions, clustering and cell type identification.

In this study, we introduce a pipeline that integrates spatial transcriptomics (ST), spatial proteomics (SP) and histology within the same lung cancer tissue section (Chong et al., 2024). Besides the innovative wet-lab approach, an integration of the data generated is made possible through an analysis pipeline in Weave software (Zhang W. et al., 2024) which allows us to register, visualize and align different spatial omics readouts, now demonstrated at the single-cell level. By co-registering these data modalities, we open a new way for studying cell segmentation, assessing the correlation between gene expression and protein abundance within the same cell, performing cell clustering and cell type classification, and gaining a more holistic understanding of tissue organization. Through this integrated approach illustrated in Figure 1, we aim to address current gaps in spatial multi-omics, demonstrating its potential to enhance multimodal analysis. Furthermore, we apply this framework to compare two lung cancer samples with distinct immunotherapy outcomes. By examining immune cell population within tumour regions, we explore how combined spatial transcriptomic and proteomic signatures may reveal key differences in the tumour-immune microenvironment.

## 2 Materials and methods

### 2.1 Sample collection and preparation

This study used formalin-fixed paraffin-embedded tissue sections from two lung carcinoma samples, obtained with patient consent (Agency for Science, Technology and Research; IRB number 2021-188). Both samples were from patients who had undergone immunotherapy. Sample A exhibited progressive disease, while sample B showed partial response.

### 2.2 Spatial transcriptomics

Tissue sections (5 µm) underwent Xenium *In Situ* Gene Expression following manufacturer’s instructions (10x Genomics, Pleasanton, CA, United States, Document CG000582 Rev E). A 289 gene human lung cancer panel was used. Sections were placed on Xenium slides within a 12 mm × 24 mm reaction region, then mounted with a cassette. After deparaffinization and decrosslinking, DNA probes were added for hybridization to target RNA sequences, followed by ligation and amplification of gene-specific barcodes. Finally, slides were loaded into the Xenium Analyzer with Xenium *In Situ* Gene Expression Reagents v1, where cycles of probe hybridization, imaging, and removal generated optical signatures for each barcode.

### 2.3 Spatial proteomics

Following Xenium, the slides underwent hyperplex immunohistochemistry (hIHC) using the COMET (Lunaphore Technologies, Switzerland). The slides underwent heat-induced epitope retrieval (HIER) with the PT module (Epredia, United States) before being mounted with microfluidic chips with an acquisition region of 9 mm × 9 mm. Sequential immunofluorescence staining was performed using off-the-shelf primary antibodies for 40 markers (Supplementary Table S1), fluorophore-conjugated secondary antibodies, and DAPI counterstain (Thermo Fisher Scientific, United States) (Rivest et al., 2023; Migliozzi et al., 2019). The COMET conducted cyclical staining, imaging, and elution, generating a final stacked fluorescence image with 41 channels, including DAPI. Background subtraction was performed (Horizon, v2.2.0.1, Lunaphore Technologies), before exporting the image for analysis. Only one ROI per sample was captured.

In parallel, serial tissue sections underwent hIHC, following the same HIER and COMET staining protocol for control (Figure 1A). The same exposure values and imaging channels (FITC and Cy5) were applied for fair comparison.

### 2.4 Hematoxylin and eosin staining

Manual hematoxylin and eosin (H&E) staining was conducted on the post-Xenium post-COMET sections, and the serial post-COMET tissue sections. The slides were imaged (Zeiss Axioscan 7, Zeiss, Germany). Manual pathology annotation was conducted on the digitized H&E images in QuPath before integration to Weave.

### 2.5 Cell segmentation

Cell segmentation was performed separately for the Xenium and COMET datasets. For Xenium, cell segmentation was based on DAPI nuclear expansion (Cook et al., 2023; Salas et al., 2025) provided by the 10x Genomics pipeline. For COMET data, CellSAM (Israel et al., 2023), a deep learning-based method integrating both nuclear (DAPI) and membrane (pan cytokeratin (PanCK)) markers, was used for segmentation. Afterwards, cells from the two segmentation methods were matched to compare their morphological and molecular features.

### 2.6 Spatially-resolved multi-omics data integration

Proteomic and transcriptomic dataset integration was conducted using Weave (version 1.0, Aspect Analytics, Genk, Belgium): DAPI images from corresponding Xenium and COMET acquisitions were co-registered to the H&E image using an automatic, non-rigid spline-based algorithm (Supplementary Figure S2A). By applying a cell segmentation mask, we calculated the mean intensity of each COMET marker and transcript count per gene per cell, generating an integrated dataset of gene and protein expression within the same cells (Figure 1B). An interactive web-based visualization was created in Weave, incorporating full resolution H&E microscopy images with pathology annotations, COMET images, Xenium gene transcripts, and respective cell segmentation results (Figure 1C).

### 2.7 Correlation of gene and protein expression

As Xenium, COMET, and Axioscan acquisition regions varied in size, unmatched pixels across the three datasets were excluded from downstream multimodal analysis. Spearman correlation between transcript count and mean immunofluorescence intensity was assessed using SciPy (Virtanen et al., 2020) for both of the aforementioned cell segmentation approaches. Among all molecular markers, 27 genes have corresponding protein markers, enabling correlation analysis.

### 2.8 Dimension reduction and clustering

Louvain clustering was employed. Cells with a total count <20 were excluded, followed by normalization of the gene expression data (e.g., total count normalization and log transformation). Dimensionality reduction (via UMAP) and neighbour graph construction (using 15 nearest neighbours and cosine similarity) was applied to create a graph where nodes (cells) are connected based on gene expression similarity (Salas et al., 2025).

### 2.9 Proteomics cell annotation

Threshold selection for each marker was visually determined using HALO v3.6 (Indica Labs, Albuquerque, NM, United States) software using raw intensity. Cells with DAPI intensity lower than the determined threshold were removed. The identified gates for each marker were subsequently used to rescale the single-cell data between 0 and 1, such that values above 0.5 identify cells that express the marker and *vice versa*. Cell types were determined using a hierarchical gating strategy (Supplementary Table S2), beginning with broad markers to categorize cells into major groups, e.g., tumour based on PanCK expression, and immune cells via CD45 expression. This was then refined into specific subpopulations (e.g., CD4^+^ T cells, CD20^+^ B cells), following the methodology described by Nirmal et al. (2022).

### 2.10 Transcriptomics cell annotation

To annotate cell types from the Xenium transcriptomic data, we used scArches—a transfer learning framework that maps single-cell profiles onto the Human Lung Cell Atlas (HLCA), which includes 61 annotated lung cell types from over 107 individuals (Sikkema et al., 2023). scArches has been applied for Xenium-based cell type annotation in several recent studies (Chen et al., 2024; Ergen and Yosef, 2023). Using the pre-trained HLCA scArches model, we performed label transfer by jointly embedding the reference and query datasets and assigning cell type labels with a k-nearest neighbours (kNN) classifier based on proximity in the latent space. We selected Level 3 annotations from HLCA’s five-tier hierarchy and compared immune cell assignments with proteomics-based cell typing.

## 3 Results

### 3.1 Cell segmentation on spatial transcriptomics and spatial proteomics

Before the evaluation of segmentation masks, the fluorescence signals of the post-Xenium COMET section were first visually compared to the fluorescence signals of the serial section that directly underwent COMET staining. Visual comparison of fluorescence signals between the two sections indicated that no aberrant staining occurred on the post-Xenium COMET slide (Supplementary Figure S1). Gross morphology also remained consistent across the two sections.

**FIGURE 2:**
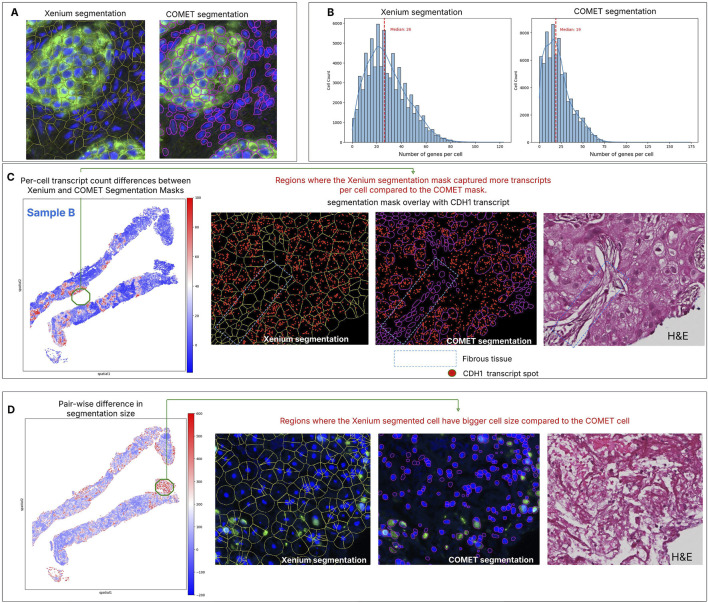
Comparison of cell segmentation on spatial transcriptomics and spatial proteomics on Sample B. **(A)** Acquisition of two modalities in the same section allows the comparison between two segmentation masks, one based on nuclear expansion and the other based on membrane protein markers. Visual assessment shows that both segmentation methods accurately capture cells with clear nuclear signals (DAPI), but with distinct morphological differences. Xenium segmentation, which uses nuclear extension, often over-expands in peripheral tissue regions, whereas COMET segmentation results in smaller cell areas and hence having more gap space between cells. **(B)** The distribution of genes per cell is higher in Xenium segmentation compared to COMET segmentation, with the latter producing more cells with low transcript counts. **(C)** Matching pairs of cells from two segmentation methods—based on nearest centroid distance—helps identify regions with the greatest discrepancies between the approaches (red area). For Sample B, cells with the largest differences in transcript spot counts are located within tumour regions. Closer inspection using CDH1 transcripts (one of the genes highly expressed in this area) reveals that these discrepancies occur in the surrounding fibrous tissue, where COMET segmentation consistently captures fewer transcript spots compared to Xenium. This comparative approach offers valuable insights into segmentation performance by leveraging both spatial transcriptomics and protein marker data from the same tissue section. **(D)** Due to the differing methods of estimating the cell boundary, differences in cell size can also be detected, especially at the edges of the tissue section or in regions with sparse cellular density.

After dataset registration, two segmentation masks were evaluated - nuclear boundary expansion provided by onboard Xenium data analysis, while the second leverages dedicated protein marker channels in the COMET data (Figure 2A). A comparison of key metrics is presented in Supplementary Table S3. Nuclear expansion identified more cells and yielded a higher median number of detected genes per cell (Figure 2B; Supplementary Figure S2B). Segmentation based on proteomics resulted in a higher proportion of cells with no captured transcripts (dropouts). This may be attributed to consistently smaller cell sizes from COMET-based segmentation compared to nuclear expansion. All cells without detected transcript spots were excluded from further analyses.

Since both cell segmentation results originated from the same tissue section, we quantitatively compared cell morphology, gene expression, and protein intensity across aligned segmentation results to assess discrepancies and identify potential outliers. Sample B is shown for illustration. The total number of transcripts detected per cell differed between the two methods, particularly in tumour areas (Figure 2C). Looking at a specific gene, CDH1 is highly expressed in tumour, but many transcripts were also detected in connective tissue, whereas the COMET segmentation identified fewer cells compared to Xenium. In fibrous tissue, the Xenium segmentation consistently produced larger cells, leading to detection of more transcripts than COMET segmentation. Conversely, cells showing pronounced morphological differences are mostly observed at tissue section edges or regions with sparse cellular density (Figure 2D). In these areas, Xenium segmentation often overestimated cell size relative to the morphology seen in the corresponding H&E while COMET segmentation produced smaller cells due to the weak or absent membrane marker signal in these regions. Similar observations were made for sample A (Supplementary Figure S2C).

Significant discrepancies between the two segmentation methods can also be assessed at the gene level, as different cell delineation impacts transcript distribution. Gene-wise analysis showed that all genes had a correlation above 0.3 between the two segmentation approaches (Supplementary Figure S3). Notably, genes with the lowest correlation (e.g., CD70, TCL1A, ASCL1) tend to have fewer total transcripts and higher dropout rates (this effect is more pronounced in COMET segmentation).

### 3.2 Correlation between transcriptomics and proteomics expression

**FIGURE 3:**
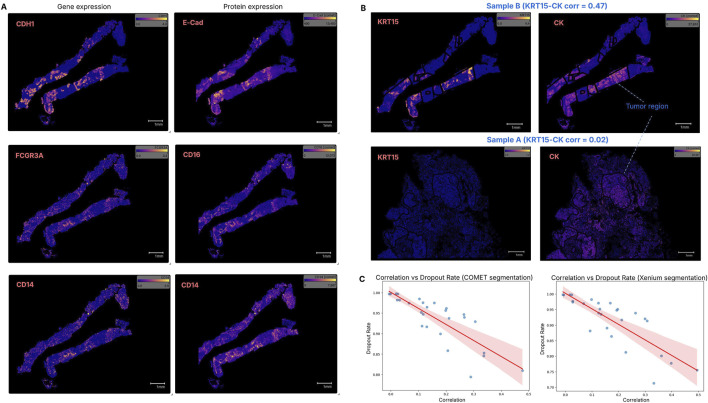
Correlation between gene expression (transcript count) and protein expression (fluorescence intensity) in the same tissue space. **(A)** The transcript–protein marker pairs CDH1–E-Cadherin, FCGR3A–CD16 and CD14–CD14 show correlation scores above 0.3 (the highest) in both Sample A and Sample B, with Sample B shown here as a representative. **(B)** The KRT15–Cytokeratin pair exhibited a high correlation in Sample B, but a very low correlation in Sample A. One possible reason could be that the pan cytokeratin protein encompasses more than the two keratin genes in the Xenium panel, thus the gene expression level had low correspondence to the protein. **(C)** Genes with higher dropout rates generally displayed lower transcript–protein correlations. Both cell segmentation methods suggest that the dropout rate negatively impacts the correlation.

After assigning a cell segmentation mask, we measured the correlation between ST and SP expression. Spearman correlation was calculated for 27 gene-protein pairs, with results summarized in Supplementary Table S4. Four transcript–protein pairs had correlation scores above 0.3 in both Sample A and B (Figure 3A): CDH1–E-Cadherin, FCGR3A–CD16, CD14–CD14, and CD68–CD68. The KRT15–Cytokeratin pair showed high correlation in Sample B (0.475) but low in Sample A (0.024), likely due to the anti-pan-cytokeratin antibody detecting more than the products of the two keratin genes in the Xenium panel (Figure 3B). Members of the S100 protein family, known prognostic markers associated with immune infiltration in some cancers (Liang et al., 2024; Chen et al., 2021), were found to exhibit consistently low correlation with their RNA transcripts.

The overall ST-SP correlation ranged from 0.0 to 0.5, indicating that some protein expressions do not correspond with their gene expression. This low ST-SP correlation is consistent with previous studies (Payne, 2015; Vogel and Marcotte, 2012) and aligns with the reported quantitative proteome map of the human body (Jiang et al., 2020). Additionally, we observed that genes with higher dropout rates—the proportion of cells in which a gene was undetected—tended to exhibit lower transcript-protein correlation, highlighting the impact of transcript detection efficiency on multi-omics integration. Both cell segmentations suggest that the dropout rate negatively affects the correlation, meaning that when fewer transcripts are detected (higher dropout), the agreement between transcript-protein marker expression becomes weaker (Figure 3C).

To investigate regional differences in ST and SP correlation across tissue types, we performed correlation analysis within pathologist-annotated tumour and non-tumour regions for both segmentations. Differential correlation analysis was applied, and Fisher’s Z-test was used to compare correlation patterns (p < 0.05). CDH1-E-Cad, KRT7/15-CK, MKI67-Ki67 are ST-SP pairs with stronger correlation in tumour regions of both samples (Supplementary Table S5). These findings are not unexpected given that cytokeratins are highly expressed in many carcinomas and Ki-67 is a marker of proliferation.

### 3.3 Dimensionality reduction and cell typing

Dimensionality reduction (DR) and cell clustering were performed on three sets: (1): ST expression, (2), SP expression, and (3) combined ST-SP data. Due to the limited number of SP markers, the reduced dimensional representation is biased toward the ST components (Supplementary Figure S4). This is expected, as the SP data contributes minimally to the overall variance structure, thereby exerting limited influence on the principal components or manifold learned during DR. Effective integration still requires computational methods that can appropriately weight and harmonize the ST and SP data, ensuring that both contribute meaningfully to downstream analyses like clustering.

Here, we take a different approach by independently annotating each modality and comparing results, with a focus on immune populations. Manual cell classification was performed using protein expression gating and visually validated using co-registered H&E-stained sections. These annotations served as high-confidence references for comparison with transcriptome-based cell typing. For Xenium, we mapped cell embeddings onto the HLCA which contains 584,944 annotated single-cell embeddings. This was used for several reasons: 1) reference and query datasets are derived from the same tissue type, 2) annotated single-cell embeddings having high percentage of immune population, and 3) in our dataset, 201 out of the 289 assayed genes overlap with the 2,000 genes used in the scArches pre-trained HLCA model.

**FIGURE 4:**
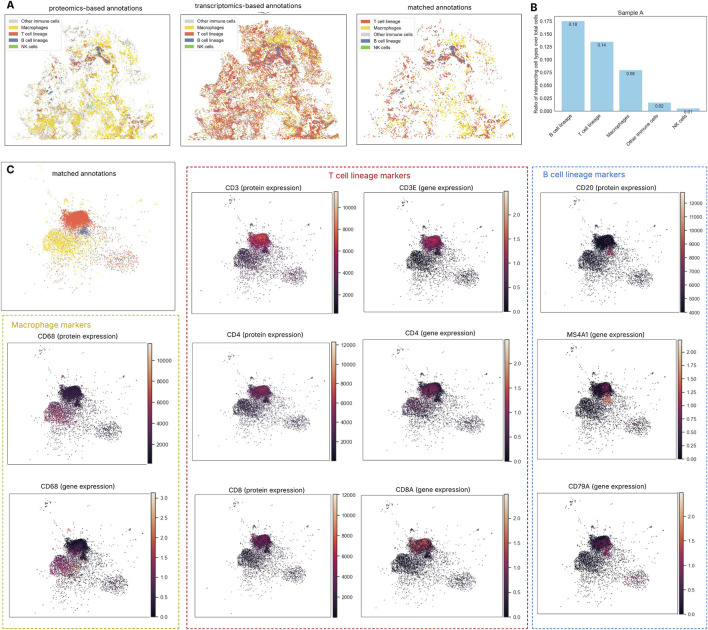
Cell type annotation from spatial transcriptomics (ST) and spatial proteomics (SP). **(A)** Concordance of immune cell annotations between ST and SP in sample A: The comparison between transcriptomics- and proteomics-based cell typing shows modest overall alignment, indicating that cell identities inferred from each modality do not always match. ST-based annotation using scRNA reference overestimates T cell abundance, potentially due to the small size and low RNA content of lymphocytes, which limits detection sensitivity. **(B)** The Jaccard index measures the similarity between immune cell groups identified by the two methods by comparing the number of overlapping cells to the total number identified by either method (union). Among immune populations, B cells show the highest agreement between methods, followed by T cells and macrophages, while NK cells display the least concordance likely due to the limited number of NK cells detected in both modalities. **(C)** UMAP-based visualization of cell annotations demonstrated that the matched B cell population (blue cluster) was characterized by high expression of both CD20 protein and the MS4A1 gene. Likewise, CD3, CD4, and CD8 markers aligned with the T cell population identified by both annotation methods, while macrophage groups were also confirmed through consistent expression of CD68 at both the transcriptomic and protein levels. This integrated view of gene and protein markers within the same cells was made possible by our multi-omics integration pipeline.

Several technical considerations were addressed when comparing cell type annotations across modalities. A key advantage of using the HLCA is its integration with the Human Cell Ontology (CL IDs), providing a standardized framework for cross-platform comparisons. However, this ontology does not include a “cancer cell” category, which appears in proteomics-based annotations. Instead, HLCA defines granular lung subtypes (e.g., Basal cells, Fibroblasts), many of which cannot be reliably distinguished using our limited COMET marker panel (Supplementary Figure S5A,B). To reconcile this, we used level 3 HLCA annotations, which group cells into broader categories with higher certainty (Lotfollahi et al., 2022). These broader categories were then mapped to the proteomics-derived classifications (Figure 4A; Supplementary Figure S5C). Secondly, we focused comparisons on immune populations, which are the primary targets of the COMET protein panel. This map enabled comparison at a higher-level resolution, which, while less granular, is appropriate for the purposes of this proof-of-concept analysis.

B cell lineage showed the highest overlap across modalities (Jaccard index: Sample B – 0.336, Sample A – 0.176), followed by T cells and macrophages; NK cells had the lowest agreement (Sample B – 0.013, Sample A – 0.006) (Figure 4B; Supplementary Figure S6A). Despite lower absolute counts, B cell lineage showed the greatest agreement across modalities in both samples. In contrast, ST-based annotation appeared to overestimate T cell numbers, as noted in a recent study where Xenium showed concentrated lymphocyte marker regions; this overrepresentation may partly result from lymphocytes’ small size and low RNA content, which affect detection sensitivity (Cook et al., 2023). Overall, the alignment between proteomics- and transcriptomics-based cell typing remains modest, suggesting that identities inferred from one modality may not fully correspond. Nevertheless, when visualized using UMAP dimensionality reduction incorporating both gene and protein expression, cells with concordant annotations from both approaches showed strong alignment with their molecular markers (Figure 4C).

## 4 Discussion

This study demonstrated an integrated experimental pipeline and analysis of spatial transcriptomics, spatial proteomics and histology at the single cell level. While previous studies have measured gene and protein expressions in the same tissue section, these were achieved with traditional methods that allow detection of few targets at a time (Hirschmann et al., 2012; Nehme et al., 2011). Newer spatial omics technologies detect targets at subcellular resolutions and integrate high-plex, automated and highly quantitative spatial profiling, granting deeper investigations into complex relationships between cells and their neighbourhoods (Rivest et al., 2023; Zhang Y. et al., 2024; Salas et al., 2025). As spatial technologies are conventionally a single omics type, two or more assays are required to obtain multi-omics data. Integration of wet lab processes on a single tissue section necessitated trials for compatibility. We have shown that tissue sections could undergo consecutive processes in two separate spatial technology machines followed by histological staining without compromising data quality or cellular morphology. One limitation is that changes in fluorescence signal in the post-Xenium COMET data could only be benchmarked against a serial section, preventing direct fluorescence intensity comparison.

As the generated data is collected separately in different formats, harmonization between the modalities is required for co-visualization and analysis. Although using the same tissue section across the pipelines reduces variations compared to serial sections, challenges are still encountered when conducting image registration (Wang et al., 2014), such as different acquisition region sizes and image resolutions between the three technologies. Given that the image acquisition window of COMET was smaller than that of Xenium and Axioscan, there was comparatively less protein data utilised in the analysis. Another complexity is non-rigid tissue deformations due to the tissues being subjected to several processes and temperatures. Nonetheless, accurate co-registration and alignment were achieved in the tissue areas captured across the transcriptomic, proteomic and the H&E images. These steps facilitated the downstream pipeline of analysing protein and transcript at single cell resolution, with tissue region-specific context.

Cell segmentation is critical, as inaccuracies in identifying cells and their boundaries can directly impact gene and protein quantification. There is no universally accepted “best” segmentation method as results often vary depending on the tissue type and segmentation algorithm used. Ultimately, the goal is to achieve segmentations that accurately reflect true cell boundaries and cell type annotation. We compared two approaches to showcase the added value of integrating proteomics. The results indicated that, for this dataset, the choice of segmentation method has minimal impact on the correlation between ST and SP across both samples. This may be due to spatial proteomics being calculated as the mean intensity per cell pixel, reducing sensitivity to variations in cell size that could arise from different segmentation methods, and the limited number of COMET markers available for direct comparison. Of the evaluated methods, nucleus expansion performs well in dense tissues but can over-assign transcripts due to inflated boundaries (Salas et al., 2025). Spatial proteomics, by contrast, captures cell shape more accurately and allows post-experiment selection of multiple segmentation markers to improve accuracy. However, membrane-based segmentation is limited by its reliance on strong, consistent expression of membrane proteins, which may not be uniform across all cells (Dayao et al., 2022). Therefore, combining transcriptomic and proteomic data in the same section enables identification of segmentation artifacts for quality control. Future work will focus on mutually exclusive gene pairs (genes rarely co-expressed in the same cell) (Hartman and Satija, 2024) to directly assess cell detection accuracy in lung tissue.

We also demonstrated that protein-gene concordance could be assessed in the same cells across different experiments. The relatively low overall ST-SP correlation (0.0–0.5) suggests that some protein expressions do not align with their respective genes, consistent with previous findings where transcript and protein enrichment diverge, possibly due to post-transcriptional and post-translational regulation (Payne, 2015; Vogel and Marcotte, 2012; Jiang et al., 2020; He et al., 2023). Our analysis further showed that concordance may vary by tissue region. For example, MS4A1–CD20 showed strong correlation in non-tumour areas of Sample B, but was more pronounced in the tumour region of Sample A (Supplementary Table S5). This observation merits further investigation, as CD20^+^ B cells are known components of the tumour microenvironment and may reflect inter-tumour heterogeneity. Moreover, MS4A1 encodes four transcript variants (V1–V4) expressed at different B-cell differentiation stages; some isoforms are translation-deficient, potentially explaining RNA–protein discordance (Nielsen et al., 2012; Zhang et al., 2023). Furthermore, we observed that the RNA-protein correlation varies across tissue regions and reflects underlying biology. For example, the MKI67-Ki67 gene-protein pair shows higher correlation in tumour areas, which is consistent with its role as a marker of cell proliferation. However, both Ki-67 mRNA and protein levels are tightly regulated by the cell cycle (Ki-67 is expressed during active proliferation and is rapidly degraded during the G1 phase or upon cell cycle exit) (Uxa et al., 2021). As a result, the asynchronous nature of cell states within tissue and the snapshot nature of transcriptomic and proteomic profiling can obscure the relationship between RNA and protein levels. This helps explain the overall low linear correlation despite the clear biological relevance of these markers. Next, the S100 protein family exhibits low RNA-protein correlation, likely due to technical and biological reasons. The Xenium panel includes only two transcripts (S100A7 and S100A12), while the antibody used can detect multiple S100 protein members. Moreover, the expression of S100 proteins is heterogeneous and cancer-type specific. For instance, S100A12 generally shows low expression in lung cancer, while S100A7 is more specific to squamous and large cell carcinomas, but not adenocarcinomas or small cell lung cancer (Liang et al., 2024). These discrepancies underscore the complexity of interpreting correlation values without broader cohort and sample size.

While same-cell transcript–protein correlations can now be explored, downstream cell type annotation remains a significant challenge due to the limited number of available gene and protein markers, particularly in imaging-based spatial omics. Yet, accurate cell identification is essential for downstream spatial analysis (e.g., differential expression, cell-cell interaction). In spatial proteomics, classification is typically done through semi-supervised methods (Barbetta et al., 2025) or manual gating based on marker intensity. However, analyzing tissue-based multiplexed imaging data poses technical challenges often requiring expert annotations and histological validation, usually resulting in broad cell type categories (Barbetta et al., 2025). In contrast, transcriptomic data benefits from numerous single-cell reference annotation tools, widely used in sequencing-based spatial transcriptomics. Still, their performance in imaging-based spatial transcriptomics has not been comprehensively evaluated (Cheng et al., 2025). One potential approach in this context is to use SP to define major cell lineages and then apply the ST gene panel for further subdivision.

Since SP and ST data were acquired on the same tissue section, a key question emerges: how do cell type annotations differ across these two platforms? Most studies rely on a single modality and do not compare transcriptomic- and proteomic-based cell labels. In our preliminary analysis comparing proteomic-based and transcriptomic-based cell labels, the CD8^+^ T cell population identified by ST was more numerous and widely distributed, whereas the same population gated in SP was much smaller and fragmented, which we posit could be attributed to protein stability (Nicolet and Wolkers, 2022). However, not all phenotypes showed such discrepancies. Manually gated B cells in SP and computationally typed B cells in ST overlapped in immune regions and aligned with the histological tissue context, validating the findings across all three modalities. Relating the ST and SP results back to H&E is crucial as H&E remains the gold standard of histopathological analysis and diagnosis (Lee et al., 2024). Although there were several limitations to cell typing, including that the gene panel used few genes overlapping with the dataset that scArches was trained on, this preliminary result shows the invaluable insight three modalities on the same section could provide. Concordant identification across modalities presents valuable opportunities. In proteomics, these cells serve as benchmarks to understand how protein combinations contribute to accurate classification. In transcriptomics, they enable gene expression imputation beyond the measured panel, enhancing Xenium resolution. For instance, results suggest the current gene panel favours B cell detection but is less effective for T cells. Follow-up studies could refine cell typing by exploring why certain lineages are better captured by one modality over another.

The limitations of this study include, but are not limited to, the small sample size of the same cancer type. Despite using two samples, this study serves as a pivot point for future studies to test a larger cohort with samples of a variety of cancer types. Our proposed wet lab methodology is applicable to all tissue types provided that the samples are prepared in accordance to histological standards and that the selected transcriptomic panel is applicable to the test samples. Though we explored a few segmentation and cell phenotyping methods, it serves as a foundation for exploration of other pipelines to increase cell typing accuracy. Moreover, the computational pipeline is modality-agnostic, assuming the molecular readouts are derived from the same section using the outlined wet lab approach, which enables robust alignment and analysis of spatial multi-omics data at the single cell level regardless of tissue or disease context. In future, the cohort could be expanded in size and cancer types, along with the integration of additional omics datasets. This study could also serve as a starting point for deeper development and standardization of the analysis pipeline, such as the preprocessing for the cell segmentation of protein markers.

## Data Availability

The datasets presented in this article are not readily available because Patient samples were used, which may include possible identifiers. Requests to access the datasets should be directed to Joe Yeong, yeongps@imcb.a-star.edu.sg.
