# Supplementary material for: An integrated approach for analyzing spatially resolved multi-omics datasets from the same tissue section
Source: Front Mol Biosci. 2025 Jul 15;12:1614288. doi: 10.3389/fmolb.2025.1614288 (PMC12304548; doi:10.3389/fmolb.2025.1614288)
Supplement: Supplementary file 1 [file Supplementaryfile1.docx]

Supplementary Material

# Supplementary Method: Image Registration Quantitative Metrics

To align COMET and Xenium images captured from the same tissue section, we used the DAPI channel present in both modalities. COMET images were registered to Xenium and transformed to Xenium’s coordinate system using three strategies: (1) Rigid + Affine (linear transformation), (2) Non-Rigid (non-linear transformation), and (3) Rigid + Non-Rigid (combined, used in the manuscript). COMET images were resampled to match Xenium's pixel spacing ([0.2300, 0.2300] µm vs. [0.2125, 0.2125] µm) for consistency in resolution and image dimensions.

## Nucleus Segmentation

In the manuscript, CellSAM and nuclear expansion were used for whole-cell segmentation. However, the resulting masks from these two methods differed substantially, making them unsuitable for evaluating registration alignment. To address this, we implemented a commonly used nuclear segmentation method, StarDist, specifically for segmenting the nuclei in DAPI-stained images and constructing evaluation metrics.

DAPI images from both platforms were segmented using the same StarDist pipeline with identical parameters. Segmentation masks enabled visual and quantitative comparison of nuclear alignment across modalities. Xenium nuclei were overlaid in red and COMET in green; overlapping nuclei appeared yellow, indicating spatial correspondence (Figure S2A1,2).

## Pairwise Cell Matching

We assumed corresponding nuclei between two modalities are spatially closer than unrelated ones. For each Xenium nucleus, the closest COMET nucleus was identified using centroid distance. A one-to-one nearest-neighbor match was retained for further analysis.

## Quantitative Evaluation Metrics

### Metric 1: Intersection-over-Union (IoU)

IoU quantifies the ratio overlap between paired segmented nuclei to union. Higher IoU values indicate better alignment between the nuclei and serve as a quantitative proxy for evaluating registration quality. We can calculate IoU in two ways:

- *Global IoU*: $\frac{Total intersection area}{Total union area}$
- *Normalized IoU*: $\frac{IoU}{\max theoretical IoU}$ for each pair of matched nuclei, where max IoU = $\frac{\min(A,B)}{max(A,B)}$ for A and B are areas of matched nucleus.

For a simple case, where one segmentation systematically produces larger or smaller nuclei than the other, a global IoU metric is good enough to quantify the overlap between two segmentation masks. However, discrepancies in segmentation mask sizes arising from processing differences between the two modalities can confound IoU measurements. For instance, if one nucleus has an area A and its counterpart has area B, the maximum achievable IoU is limited to min(A,B) and max(A,B) (max theoretical IoU). Therefore, normalized IoU accounts for segmentation size discrepancies between modalities and provides a fairer comparison. We reported the average normalized IoU across all matched pairs.

### Metric 2: Centroid Distance

In addition to IoU values, to assess the alignment quality between COMET and Xenium modalities, we could also utilize centroid distance as a quantitative metric. This involved measuring the Euclidean distance (in µm) between the centroids of matched nuclei pairs across the two modalities (Figure S2A3). A smaller average centroid distance indicates better registration accuracy.

Beyond the mean distance, analyzing the distribution of centroid distances provides deeper insights into registration performance. Specifically, we examined the proportion of matched nuclei pairs with centroid distances less than 1 µm, which reflects the precision of alignment at very high level (average nucleus diameter is approximately 7.5 µm). This comprehensive approach, considering both average distances and their distribution, offers a robust evaluation of registration quality between COMET and Xenium images.

### Performance Summary

| **Metric** | | **Rigid+Affine** | **Non-Rigid** | **Rigid+Non-Rigid (used in manuscript)** |
| --- | --- | --- | --- | --- |
| IoU | Global IoU | 0.629 | 0.646 | 0.661 |
|  | Normalized IoU (mean ± SD) | 0.75 ± 0.22 | 0.78 ± 0.20 | 0.80 ± 0.20 |
| Centroid distance | Mean Centroid Distance (µm) | 1.46 ± 1.16 | 1.35 ± 1.12 | 1.25 ± 1.10 |
|  | Matched nucleus within 1 µm (%) | 40.48% | 45.26% | 51.78% |

The registration approach presented in the manuscript (Rigid + Non-Rigid) demonstrates single-cell precision, as confirmed by visual validation. In addition, across both quantitative metrics (IoU) and centroid distance) our pipeline consistently achieves the highest registration accuracy when compared to standard linear transformations or purely non-rigid methods.

Specifically, our method yields the highest Global IoU and Normalized IoU, indicating the greatest spatial overlap between the two modalities post-registration. In terms of centroid distance, not only does our approach achieve the lowest average distance, but it also results in the highest proportion of cell pairs (52%) aligned within 1 micron, reflecting precise alignment at the single-cell level.

## Considerations and Caveats

One caveat of these two metrics is their dependence on the quality of nuclei segmentation in two different images. Variability in segmentation could potentially influence the accuracy of the registration assessment. However, by applying the same segmentation pipeline with consistent parameters across the validation process, we ensure that the comparison remains fair and systematic. Under these controlled conditions, the metrics still provide meaningful and informative insights into the quality of the registration. Additionally, unmatched nuclei, especially those detected in only one modality, can skew these metrics and lead to erroneous pairings. To address this, we implemented a filtering step based on centroid distance, excluding nucleus pairs with distances exceeding 50 pixels. This threshold, approximately twice the average nucleus diameter, helps eliminate unlikely matches and enhances the reliability of the registration evaluation.

# Supplementary Figures and Tables

## Supplementary Figures


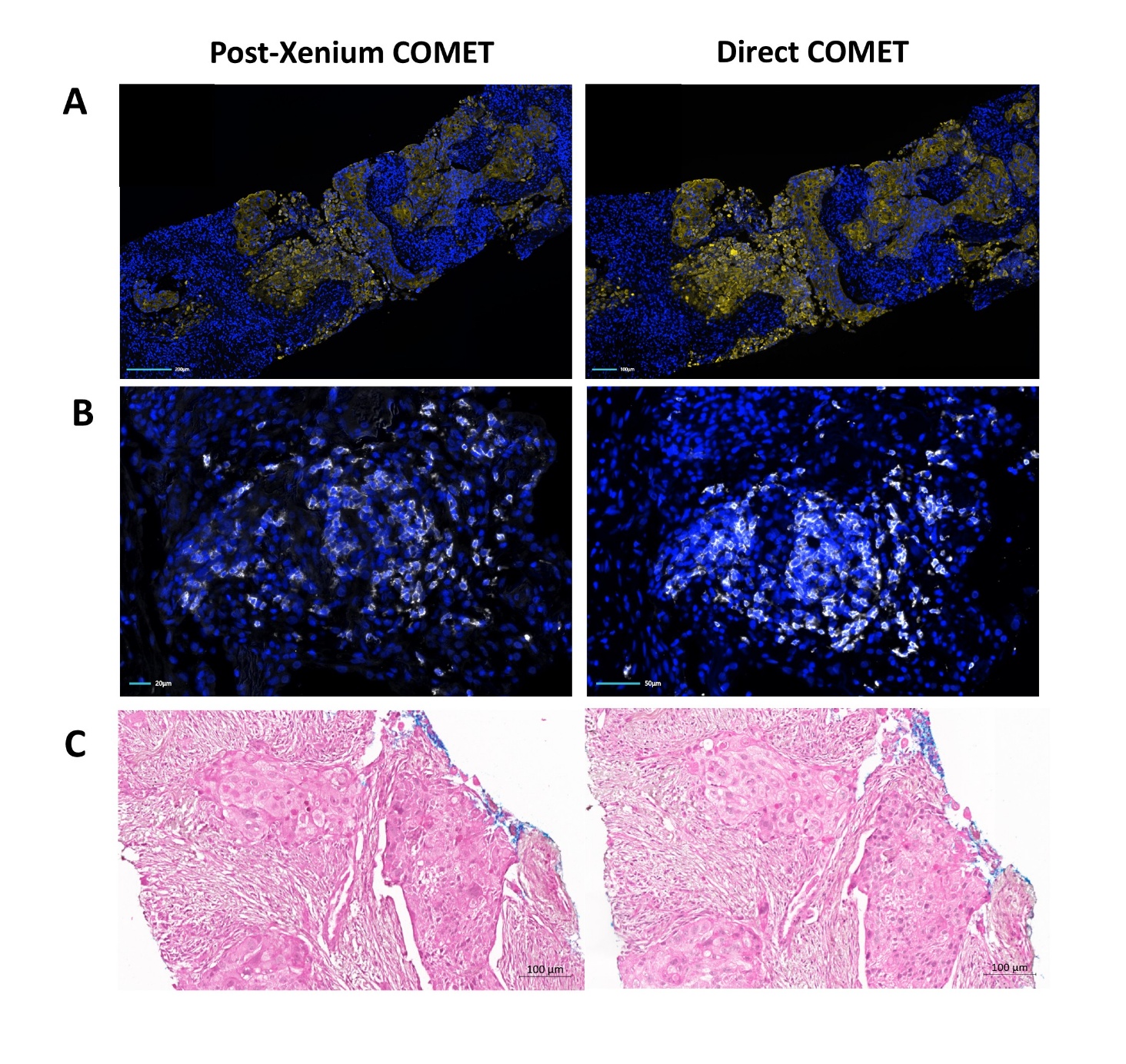


**Figure S1. Qualitative evaluation of tissue sections that underwent post-Xenium COMET and direct COMET. (A)** Using the same histogram adjustment values for fair comparison, it was observed that generally the same tumour regions in both images were identified by the PanCK stains, albeit the slightly weaker signal in the post-Xenium COMET image. **(B)** Again, using the same histogram adjustment values, it was observed that the CD20+ cells were detected in the same tissue regions, albeit the slightly weaker signal in the post-Xenium COMET image. **(C)** The two H&E images do not differ much in colour intensities and general tissue landscape.


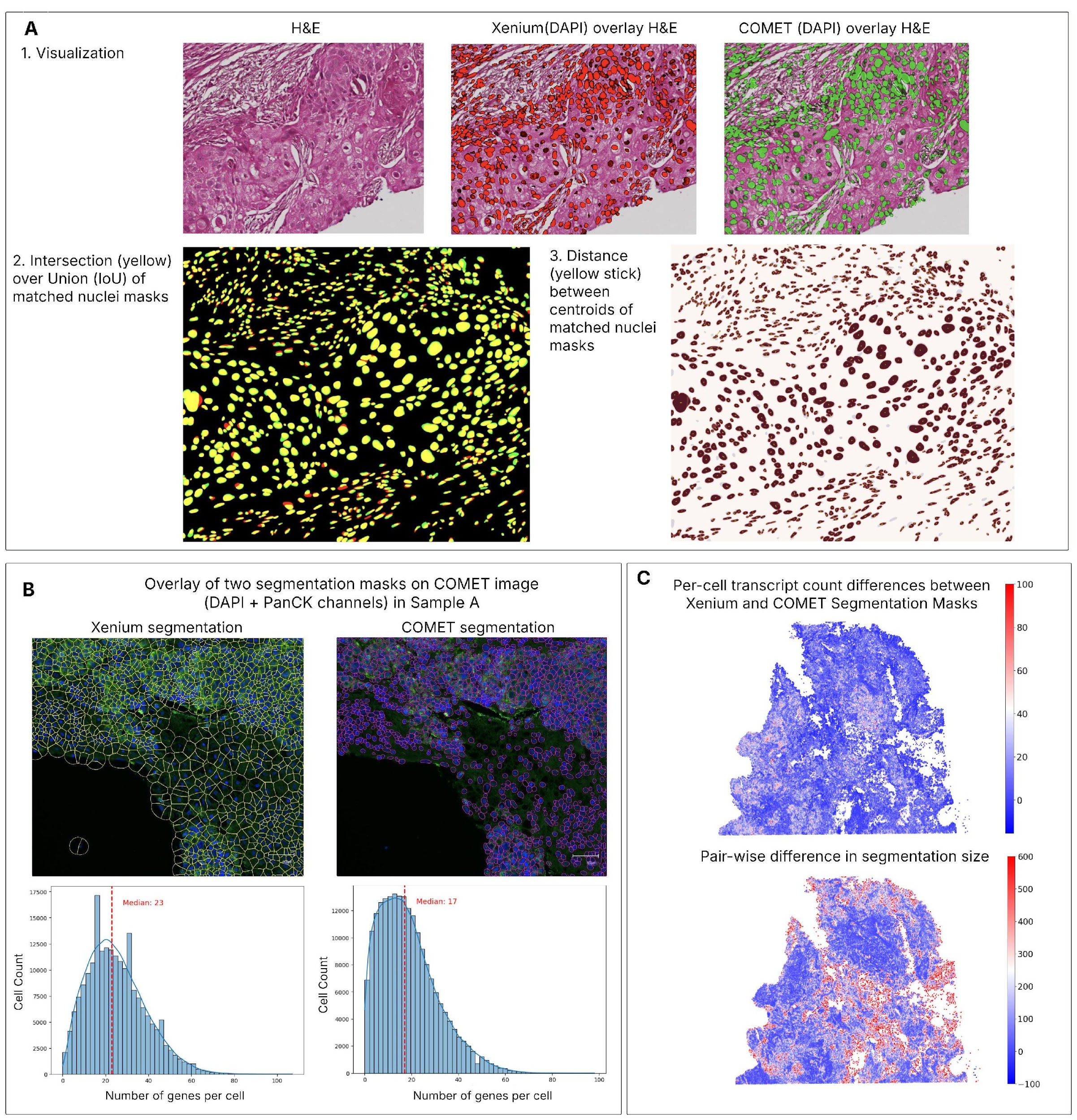


**Figure S2. Comparison of cell segmentation in spatial transcriptomics and spatial proteomics. (A)** Image registration is essential for integrating data and subsequently comparing segmentation masks at the single-cell level. Visual assessment confirmed precise alignment, particularly through the matching of DAPI signals across Xenium, COMET, and H&E nuclear staining. To quantitatively assess alignment accuracy, we employed Intersection-over-Union (IoU) and centroid distance metrics (see Supplementary Method: Image Registration Quantitative Metrics). Our evaluation demonstrated that the registration strategy used outperformed both standard linear (rigid + affine) and purely non-rigid transformations, achieving superior alignment accuracy. **(B)** The observations in Sample A mirrored those in Sample B (in Figure 2). (1) Xenium segmentation, which relies on nuclear extension, frequently over expands in peripheral tissue regions, while COMET segmentation generates smaller cell areas, resulting in wider gaps between cells. The distribution of genes per cell is higher in Xenium segmentation compared to COMET segmentation, due to the perceived larger cell areas. **(C)** Matched cell pairs from the two segmentation methods revealed differences in cell size—particularly at tissue edges or in regions of low cellular density—as well as in the total number of captured transcripts (in red).

**
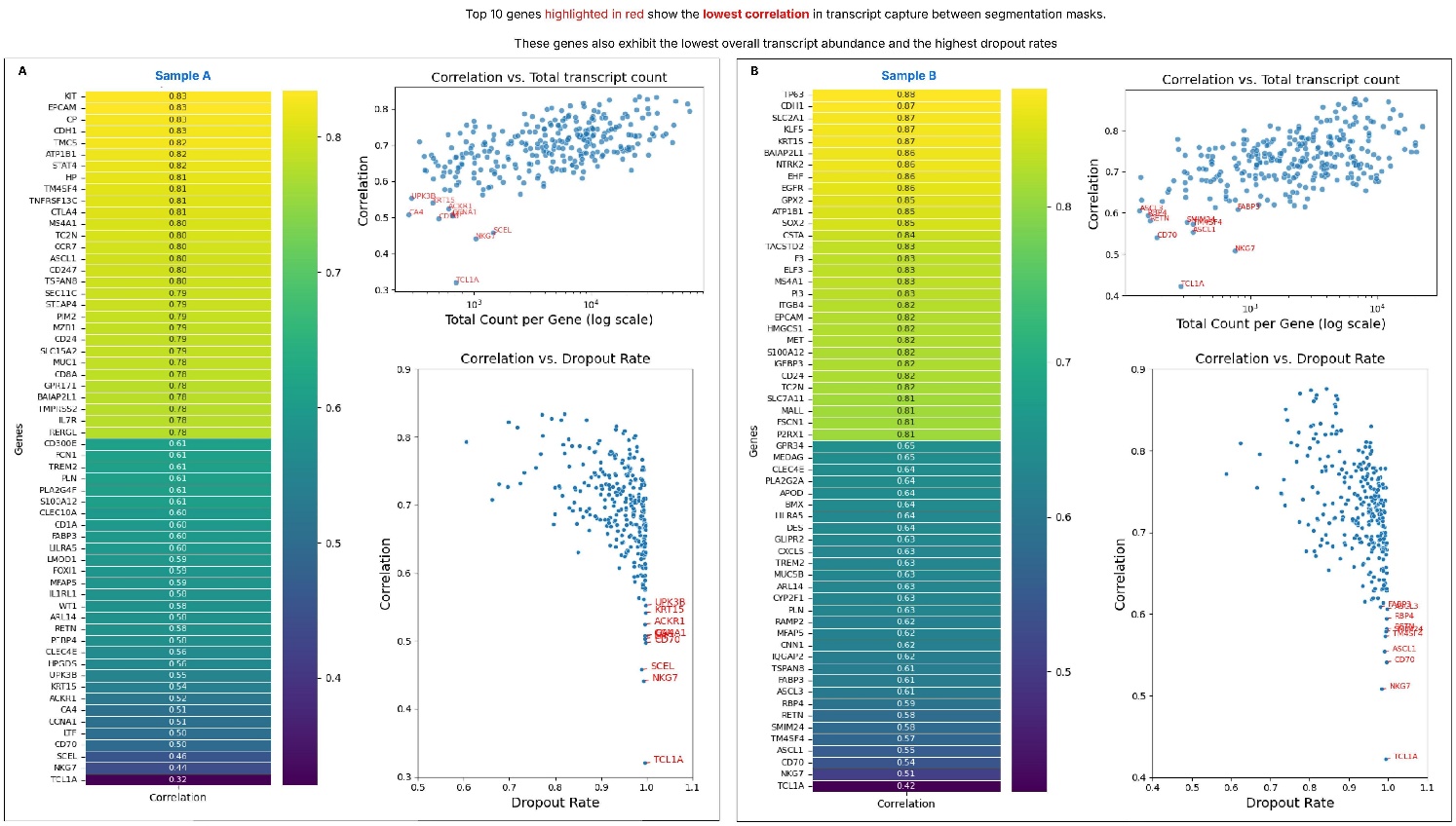
**

**Figure S3. Top 30 genes with the highest and lowest correlation of transcript counts between the two segmentation methods. (A)** Pair-wise analysis revealed that all genes had a correlation above 0.3 in Sample A. **(B)** In Sample B, pair-wise analysis revealed that all genes had a correlation above 0.4. In both samples, genes with the lowest correlation (e.g., *TCL1A, NKG7, CD70, SCEL*) were notably influenced by segmentation boundaries. These genes also tended to have lower overall transcript abundance and higher dropout rate (particularly when using the COMET segmentation), suggesting that segmentation choice disproportionately impacts low-expression genes.

**
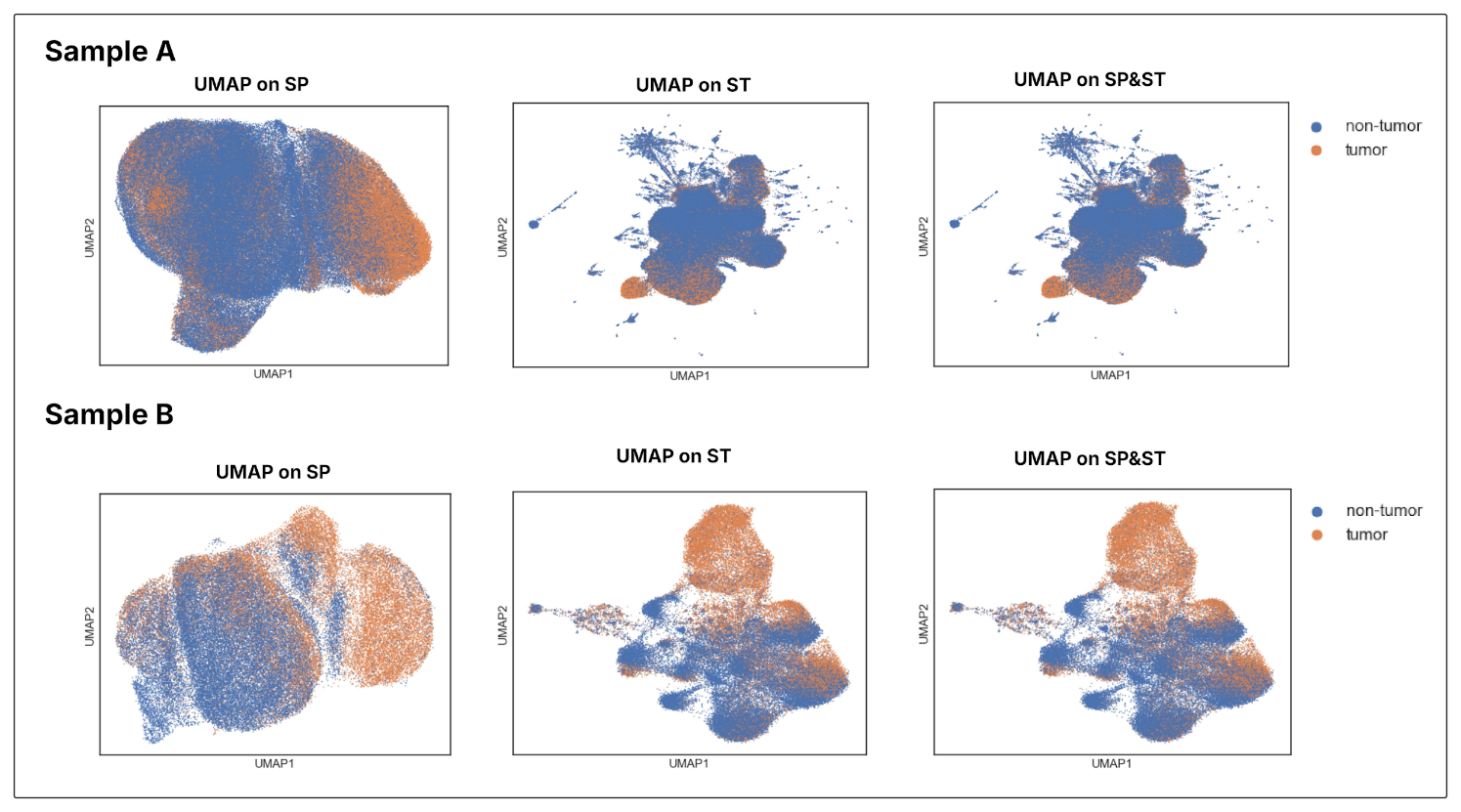
**

**Figure S4. Dimensionality reduction (DR) was performed on three sets: (1) SP expression, (2) ST expression, and (3) combined SP-ST data.** Due to the limited number of SP markers, the reduced dimensional representation is biased toward the ST components. This is expected, as the SP data contributes minimally to the overall variance structure, thereby exerting limited influence on the principal components or manifold learned during DR.


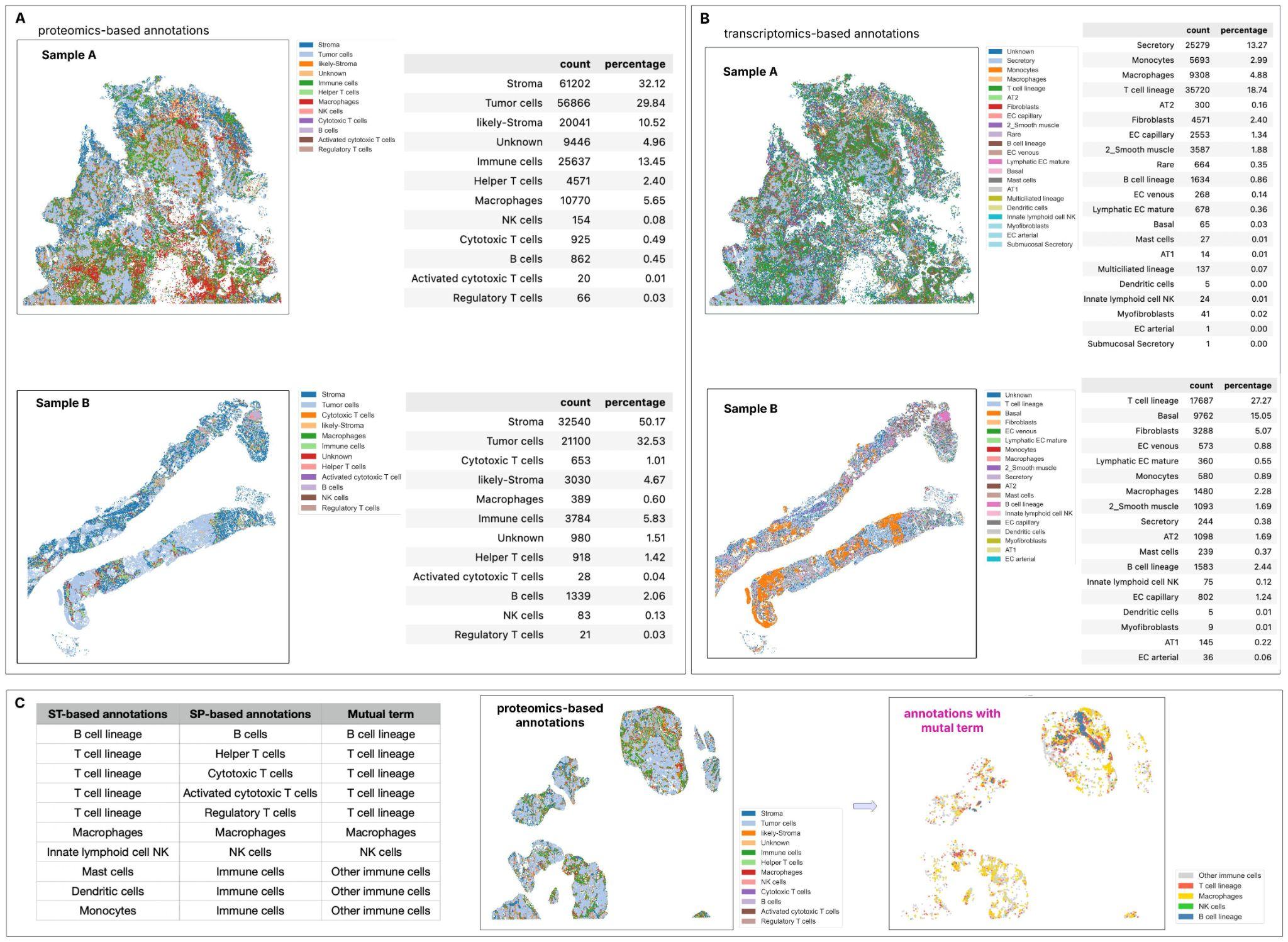


**Figure S5. Cell type annotation across whole-tissue sections from two lung samples.** **(A)** Cell typing on spatial proteomics was performed based on protein expression using a hierarchical gating strategy. The process began with broad markers to distinguish major cell groups, such as tumour cells (e.g., identified by PanCK expression) and immune cells (e.g., identified by CD45 expression). These broad categories were then further refined into specific subpopulations, including CD4⁺ T cells and CD20⁺ B cells. Gating thresholds for each marker were manually defined by domain experts. The resulting classifications were visually re-evaluated on the Weave platform, using co-registered hematoxylin and eosin (H&E) stained sections, to ensure that the identified subclusters corresponded to appropriate histological regions. Cells that are not assigned to any type are "Unknown". This multi-step validation process provided high-confidence reference annotations to compare with ST results. Tumour and stroma regions show strong alignment with the histological annotations. **(B)** Cell types predicted using a single-cell transcriptomic reference from ST, showing Level 3 annotations from the five-tier HLCA hierarchy (Level 1 = broadest, Level 5 = most granular). Each query cell got an uncertainty score that showed how confidently the label was assigned to the cell, and a cell was labelled "Unknown" when the score was higher than a certain threshold. Notably, there is a marked overrepresentation of CD8⁺ T cells in both samples based on ST-based cell typing. Both cell typing approaches are performed on protein-based segmentation. **C)** To align annotations from ST and SP, cells were grouped into broader, mutually defined categories focused on immune populations. Matched classifications were then mapped back onto the tissue to identify spatially concordant cells.


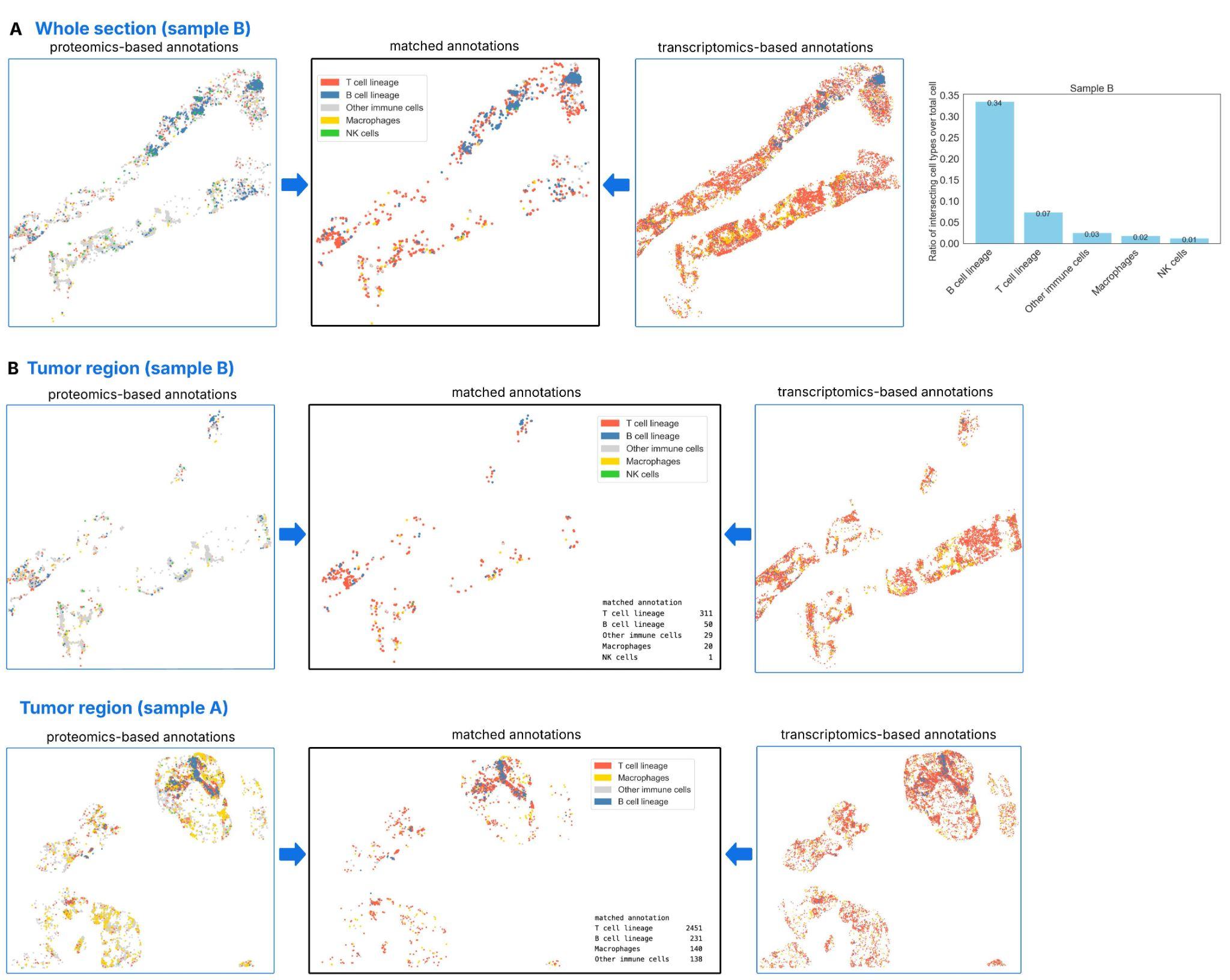


**Figure S6.** **Cell type annotation from spatial proteomics (manual gating) and spatial transcriptomics (using single-cell reference), with common matched annotations from both modalities.** **(A)** Of the five listed cell phenotypes in the entirety of Sample B, the B cell lineage had the highest ratio of common cells identified between the two modalities. Although the T cell lineage had the next highest ratio, many T cells annotated by the spatial transcriptomics pipeline did not match to the T cells annotated by the manual gating of spatial proteomics results. **(B)** Comparing the cell phenotyping within the tumour region revealed that again both T and B cell lineages had the highest matched ratios between the two cell type annotation methods.

## Supplementary Tables

**Table S1. Primary antibodies used for hyperplex immunohistochemistry (hIHC) staining.** List of primary antibodies in the 40-plex protein panel used in COMET.

| aSMA | BDCA-2 | CD3 | CD4 | CD8 |
| --- | --- | --- | --- | --- |
| CD11b | CD11c | CD14 | CD15 | CD16 |
| CD19 | CD20 | CD21 | CD31 | CD34 |
| CD38 | CD39 | CD45 | CD45RA | CD45RO |
| CD56 | CD68 | CD163 | CK | ECAD |
| EOMES | FOXP3 | GZMB | HLA-DR | ICOS |
| IDO-1 | Ki67 | LAG-3 | LaminA | PD1 |
| PD-L1 | Podoplanin | S100 | Tryptase | VISTA |

**Table S2.** **The gating scheme for spatial-proteomics markers.** Using manually defined intensity thresholds for protein markers derived from the spatial proteomics image, a hierarchical gating approach was employed to classify cell phenotypes. This process assigns labels to individual cells through a sequential probability-based classification. The algorithm operates on the assumption that true signal intensities exceed those of potential bleed-through or artifacts. For instance, if a CD20⁺ B cell is located adjacent to a CD8⁺ cytotoxic T cell, and some CD8 signal bleeds into the B cell, the algorithm compares the scaled intensities of both markers and assigns the cell as a B cell due to stronger CD20 expression. This classification follows a tree-like structure: initially dividing cells into broad categories (e.g., tumour cells via cytokeratin, or immune cells via CD45), and then progressively refining them into specific types (e.g., cytotoxic T cells, B cells), and further into finer subtypes through subsequent steps.


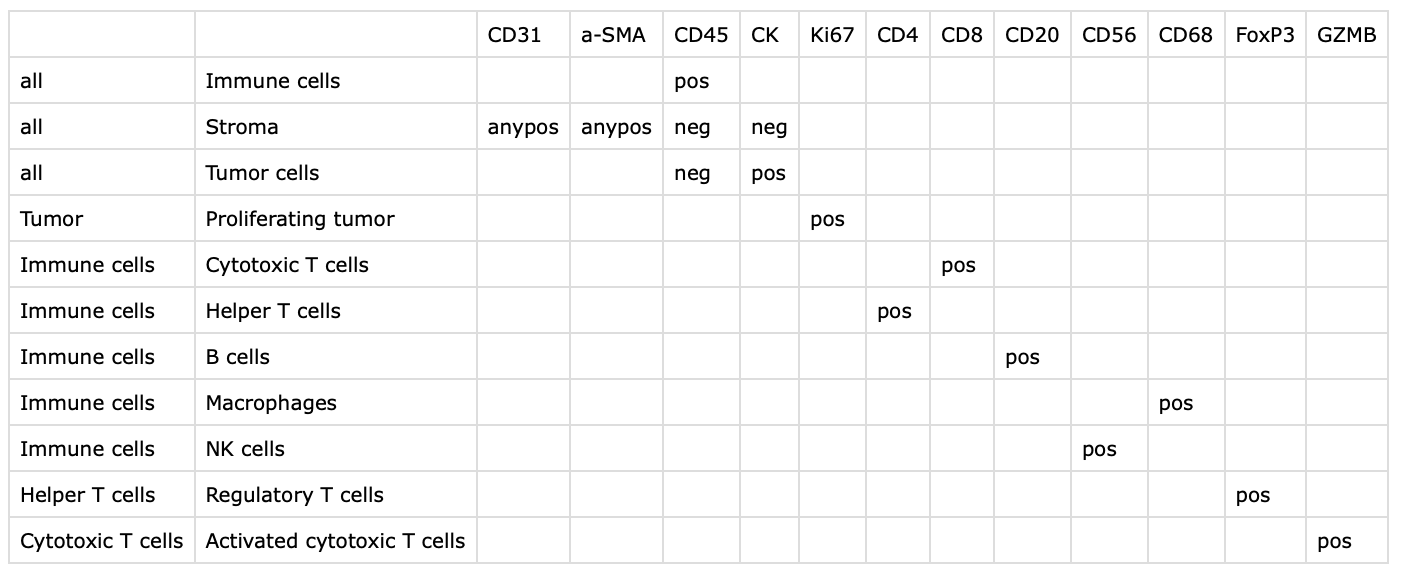


**Table S3. Comparison between COMET-based segmentation and Xenium-based segmentation**

| **Sample** |  | **Total segmented cell** | **Drop out cell ratio^a^** | **Median cell area**  **(um^2^)** | **Median total count per cell** | **Median number of genes per cell** |
| --- | --- | --- | --- | --- | --- | --- |
| **A** | Xenium-based segmentation | 238,121 | 0.0029 | 115.73 ± 123.79 | 42 | 26 |
|  | Comet-based  segmentation | 233,465 | 0.0255 | 51.52 ± 34.39 | 17 | 19 |
| **B** | Xenium-based segmentation | 77,292 | 0.0025 | 123.23 ± 121.05 | 33 | 23 |
|  | Comet-based  segmentation | 76,531 | 0.0170 | 51.05 ± 42.08 | 21 | 17 |

**^a^: number of cells that capture no transcript at all in total segmented cells**

**Table S4. Correlation of gene and protein expression in two samples A & B.** Protein markers in the spatial proteomics panel mapped to their corresponding gene available in the spatial transcriptomics panel. Spearman correlation between transcript count and mean immunofluorescence intensity was assessed using SciPy.

| **Sample A** | | | | | |
| --- | --- | --- | --- | --- | --- |
| xenium | comet | correlation (xenium-seg) | correlation (comet-seg) | dropout_rate (xenium-seg) | dropout_rate (comet-seg) |
| CDH1 | E-Cad | 0.495 | 0.477 | 0.755 | 0.810 |
| FCGR3A | CD16 | 0.398 | 0.337 | 0.778 | 0.845 |
| CD14 | CD14 | 0.361 | 0.338 | 0.801 | 0.852 |
| CD68 | CD68 | 0.332 | 0.290 | 0.713 | 0.794 |
| CD3E | CD3 | 0.307 | 0.306 | 0.913 | 0.929 |
| CD34 | CD34 | 0.299 | 0.266 | 0.920 | 0.940 |
| CD3D | CD3 | 0.264 | 0.265 | 0.938 | 0.947 |
| CD4 | CD4 | 0.227 | 0.206 | 0.813 | 0.858 |
| KRT7 | CK | 0.212 | 0.211 | 0.916 | 0.937 |
| CD8A | CD8 | 0.197 | 0.201 | 0.951 | 0.962 |
| FOXP3 | FoxP3 | 0.193 | 0.199 | 0.948 | 0.957 |
| MKI67 | Ki67 | 0.174 | 0.180 | 0.863 | 0.899 |
| MS4A1 | CD20 | 0.169 | 0.157 | 0.971 | 0.975 |
| PDPN | Podoplanin | 0.156 | 0.130 | 0.891 | 0.916 |
| CD38 | CD38 | 0.134 | 0.110 | 0.935 | 0.952 |
| GZMB | GZMB | 0.130 | 0.130 | 0.952 | 0.965 |
| ITGAM | CD11b | 0.125 | 0.117 | 0.938 | 0.947 |
| CD19 | CD19 | 0.124 | 0.116 | 0.971 | 0.975 |
| CD8B | CD8 | 0.098 | 0.104 | 0.983 | 0.985 |
| CD163 | CD163 | 0.093 | 0.113 | 0.881 | 0.918 |
| CD274 | PD-L1 | 0.069 | 0.065 | 0.970 | 0.975 |
| LAG3 | LAG-3 | 0.028 | 0.031 | 0.977 | 0.982 |
| S100B | S100 | 0.027 | 0.023 | 0.974 | 0.982 |
| KRT15 | CK | 0.024 | 0.026 | 0.998 | 0.997 |
| PDCD1 | PD1 | 0.016 | 0.020 | 0.998 | 0.997 |
| S100A12 | S100 | -0.006 | -0.001 | 0.997 | 0.997 |
| S100A7 | S100 | -0.009 | -0.006 | 0.997 | 0.997 |
| **Sample B** | | | | | |
| xenium | comet | correlation (xenium-seg) | correlation (comet-seg) | dropout_rate (xenium-seg) | dropout_rate (comet-seg) |
| CDH1 | E-Cad | 0.533 | 0.512 | 0.795 | 0.818 |
| KRT15 | CK | 0.475 | 0.449 | 0.858 | 0.875 |
| FCGR3A | CD16 | 0.457 | 0.382 | 0.753 | 0.830 |
| CD14 | CD14 | 0.425 | 0.382 | 0.771 | 0.842 |
| CD68 | CD68 | 0.309 | 0.300 | 0.709 | 0.793 |
| CD163 | CD163 | 0.300 | 0.265 | 0.824 | 0.887 |
| CD34 | CD34 | 0.298 | 0.261 | 0.912 | 0.935 |
| CD3E | CD3 | 0.283 | 0.298 | 0.902 | 0.923 |
| CD3D | CD3 | 0.273 | 0.287 | 0.917 | 0.935 |
| MS4A1 | CD20 | 0.272 | 0.249 | 0.944 | 0.956 |
| KRT7 | CK | 0.271 | 0.269 | 0.912 | 0.923 |
| CD4 | CD4 | 0.263 | 0.242 | 0.739 | 0.808 |
| CD38 | CD38 | 0.246 | 0.226 | 0.864 | 0.897 |
| FOXP3 | FoxP3 | 0.215 | 0.218 | 0.943 | 0.954 |
| CD8A | CD8 | 0.201 | 0.200 | 0.943 | 0.956 |
| MKI67 | Ki67 | 0.195 | 0.198 | 0.902 | 0.921 |
| CD19 | CD19 | 0.186 | 0.171 | 0.946 | 0.955 |
| GZMB | GZMB | 0.159 | 0.161 | 0.942 | 0.958 |
| PDPN | Podoplanin | 0.135 | 0.126 | 0.787 | 0.841 |
| CD8B | CD8 | 0.113 | 0.116 | 0.975 | 0.979 |
| CD274 | PD-L1 | 0.100 | 0.089 | 0.959 | 0.965 |
| S100A12 | S100 | 0.098 | 0.098 | 0.843 | 0.867 |
| S100A7 | S100 | 0.065 | 0.065 | 0.925 | 0.936 |
| ITGAM | CD11b | 0.053 | 0.073 | 0.901 | 0.919 |
| S100B | S100 | 0.048 | 0.044 | 0.972 | 0.980 |
| PDCD1 | PD1 | 0.036 | 0.048 | 0.994 | 0.994 |
| LAG3 | LAG-3 | 0.023 | 0.037 | 0.968 | 0.975 |

**Table S5. Correlation of gene and protein expression in Samples A and B within tumour region**

| **Sample A** | | | | | |
| --- | --- | --- | --- | --- | --- |
| xenium | comet | correlation ST - SP (tumour) | correlation ST - SP (non-tumour) | Z-score | P-value |
| **CDH1** | **E-Cad** | **0.54** | **0.31** | 57 | 0.0000 |
| **KRT7** | **CK** | **0.28** | **0.10** | 37 | 0.0000 |
| **MKI67** | **Ki67** | **0.23** | **0.13** | 20 | 0.0000 |
| CD4 | CD4 | 0.28 | 0.18 | 20 | 0.0000 |
| **MS4A1** | **CD20** | **0.22** | **0.13** | 18 | 0.0000 |
| CD8A | CD8 | 0.23 | 0.17 | 13 | 0.0000 |
| CD3E | CD3 | 0.34 | 0.28 | 13 | 0.0000 |
| CD38 | CD38 | 0.17 | 0.11 | 12 | 0.0000 |
| CD3D | CD3 | 0.29 | 0.24 | 11 | 0.0000 |
| CD19 | CD19 | 0.16 | 0.10 | 11 | 0.0000 |
| FCGR3A | CD16 | 0.41 | 0.38 | 9 | 0.0000 |
| S100B | S100 | 0.05 | 0.01 | 8 | 0.0000 |
| CD274 | PD-L1 | 0.09 | 0.06 | 6 | 0.0000 |
| CD8B | CD8 | 0.11 | 0.09 | 5 | 0.0000 |
| S100A7 | S100 | 0.00 | -0.01 | 3 | 0.0005 |
| PDCD1 | PD1 | 0.02 | 0.01 | 2 | 0.0152 |
| S100A12 | S100 | -0.00 | -0.01 | 2 | 0.1291 |
| LAG3 | LAG-3 | 0.03 | 0.03 | 1 | 0.3638 |
| KRT15 | CK | 0.02 | 0.02 | 1 | 0.3995 |
| FOXP3 | FoxP3 | 0.19 | 0.20 | -2 | 0.0304 |
| CD34 | CD34 | 0.28 | 0.30 | -5 | 0.0000 |
| CD163 | CD163 | 0.09 | 0.12 | -6 | 0.0000 |
| CD68 | CD68 | 0.30 | 0.34 | -9 | 0.0000 |
| GZMB | GZMB | 0.10 | 0.14 | -9 | 0.0000 |
| ITGAM | CD11b | 0.08 | 0.13 | -12 | 0.0000 |
| PDPN | Podoplanin | 0.09 | 0.15 | -13 | 0.0000 |
| CD14 | CD14 | 0.29 | 0.39 | -23 | 0.0000 |
| **Sample B** | | | | | |
| xenium | comet | correlation ST - SP (tumour) | correlation ST - SP (non-tumour) | Z-score | P-value |
| **CDH1** | **E-Cad** | **0.66** | **0.17** | 76 | 0.0000 |
| **KRT15** | **CK** | **0.49** | **0.16** | 46 | 0.0000 |
| **MKI67** | **Ki67** | **0.25** | **0.11** | 17 | 0.0000 |
| FCGR3A | CD16 | 0.51 | 0.41 | 15 | 0.0000 |
| CD4 | CD4 | 0.31 | 0.20 | 15 | 0.0000 |
| CD14 | CD14 | 0.46 | 0.39 | 10 | 0.0000 |
| KRT7 | CK | 0.27 | 0.20 | 9 | 0.0000 |
| S100B | S100 | 0.05 | 0.01 | 5 | 0.0000 |
| LAG3 | LAG-3 | 0.04 | 0.01 | 5 | 0.0000 |
| PDCD1 | PD1 | 0.05 | 0.02 | 4 | 0.0000 |
| CD274 | PD-L1 | 0.11 | 0.09 | 3 | 0.0008 |
| ITGAM | CD11b | 0.06 | 0.04 | 3 | 0.0054 |
| GZMB | GZMB | 0.16 | 0.15 | 1 | 0.3471 |
| FOXP3 | FoxP3 | 0.22 | 0.21 | 1 | 0.6007 |
| CD163 | CD163 | 0.30 | 0.30 | 0 | 0.8311 |
| CD68 | CD68 | 0.29 | 0.30 | -1 | 0.5566 |
| PDPN | Podoplanin | 0.14 | 0.15 | -1 | 0.1962 |
| CD3D | CD3 | 0.26 | 0.29 | -3 | 0.0062 |
| CD8B | CD8 | 0.10 | 0.13 | -4 | 0.0003 |
| CD8A | CD8 | 0.18 | 0.21 | -4 | 0.0001 |
| CD3E | CD3 | 0.26 | 0.31 | -7 | 0.0000 |
| CD38 | CD38 | 0.20 | 0.28 | -11 | 0.0000 |
| CD19 | CD19 | 0.12 | 0.20 | -11 | 0.0000 |
| S100A7 | S100 | -0.06 | 0.04 | -12 | 0.0000 |
| CD34 | CD34 | 0.21 | 0.32 | -15 | 0.0000 |
| S100A12 | S100 | -0.09 | 0.05 | -17 | 0.0000 |
| **MS4A1** | **CD20** | **0.08** | **0.37** | -38 | 0.0000 |
